# Toll-like receptor-associated keratitis and strategies for its management

**DOI:** 10.1007/s13205-015-0280-y

**Published:** 2015-02-15

**Authors:** Amandeep Kaur, Vijay Kumar, Simranjeet Singh, Joginder Singh, Niraj Upadhyay, Shivika Datta, Sourav Singla, Virender Kumar

**Affiliations:** 1 Department of Chemistry, Lovely Professional University, Phagwara, 144411 Punjab India; 2 Department of Biotechnology, Lovely Professional University, Phagwara, 144411 Punjab India; 3 Department of Zoology, Lovely Professional University, Phagwara, 144411 Punjab India

**Keywords:** Toll-like receptor, Keratitis, Infection, Treatment, Cytokines

## Abstract

Keratitis is an inflammatory condition, characterized by involvement of corneal tissues. Most recurrent challenge of keratitis is infection. Bacteria, virus, fungus and parasitic organism have potential to cause infection. TLR are an important class of protein which has a major role in innate immune response to combat with pathogens. In last past years, extensive research efforts have provided considerable abundance information regarding the role of TLR in various types of keratitis. This paper focuses to review the recent literature illustrating amoebic, bacterial, fungal and viral keratitis associated with Toll-like receptor molecules and summarize existing thoughts on pathogenesis and treatment besides future probabilities for prevention against TLR-associated keratitis.

## Introduction

**Fig. 1:**
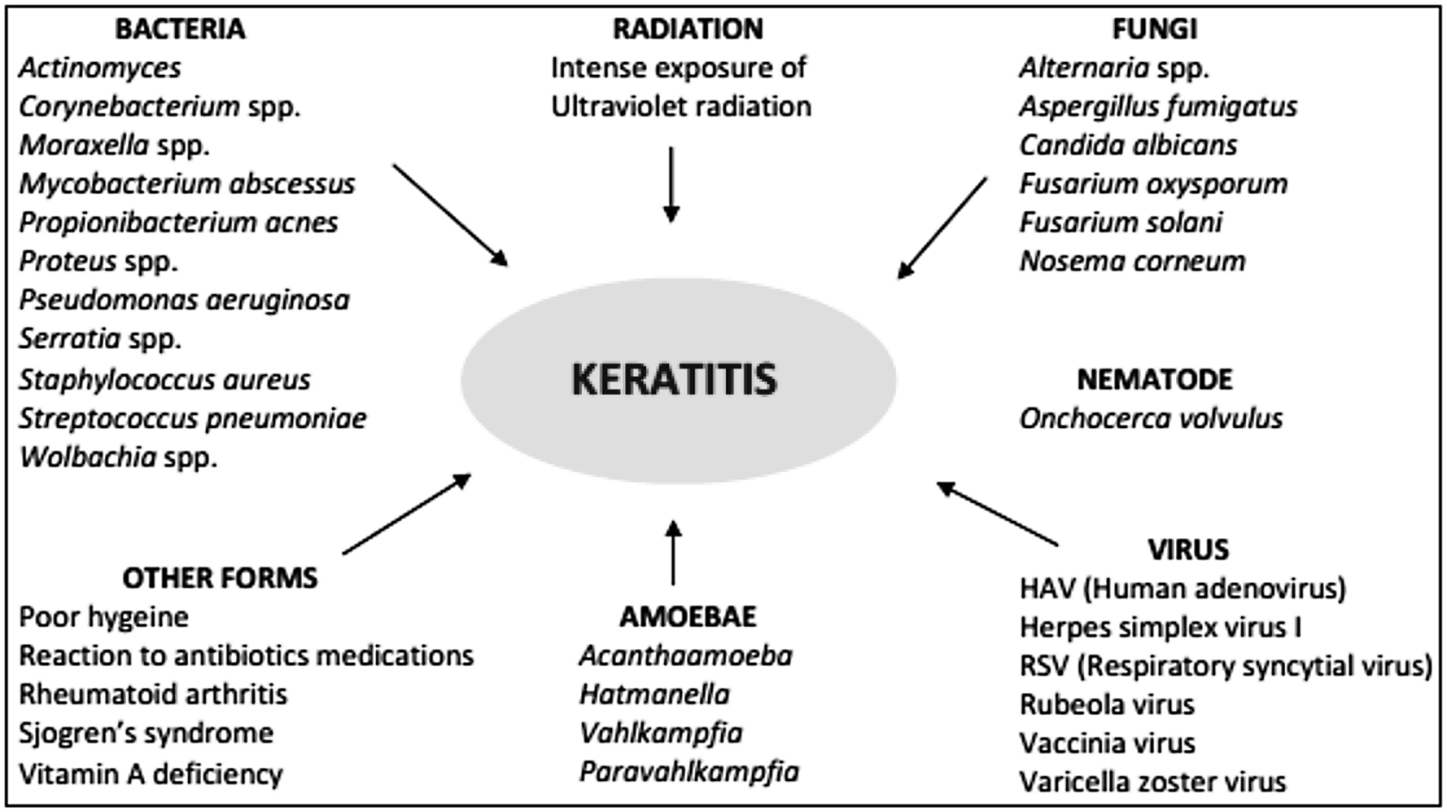
Major categories of corneal inflammation (keratitis) of eyes

Potential targets of eye are mucosal surfaces, such as the mucosal epithelium of the cornea, and interior chambers, such as the vitreous humor. Human corneal epithelial cells in eye serve as a first line of defense to protect cornea from harmful pathogens. The recognition ability of epithelial cells to detect specific pathogens is critical for the inauguration of innate and adaptive immune responses (Philpott et al. 2001). Recent researches revealed that recognition of conserved molecular motifs like pathogen-associated molecular patterns (PAMP) of microbes by TLRs expressed on cell surfaces plays an essential role in innate immunity (O’Neill 2008; Aderem and Ulevitch 2000). Due to recognition of pervading microbes may provoke the secretion of cytokines that engage inflammatory cells to kill the microbes (Johnson et al. 2006; Kumar et al. 2006a; Sun et al. 2006; Vora et al. 2003). HCEC initiates innate immune responses through discharging pro-inflammatory cytokines like IL-1 and TNF-α after triggering cell with Gram-negative bacteria (Zhang et al. 2005). When inflammatory response crosses its breaking point, it can lead to corneal epithelial cell destruction by inflammatory cell. Consequently, it can lead to blindness. Parkinson and other neurodegenerative disorders, inflammation of the cornea, have been most studied in case of exposure to neurotoxic and carcinogenic pesticides such as organophosphates, carbamates, organochlorines, pyrethroids and some other insecticides, since they interfere with neurotransmission and function of ion channels in the nervous system (Costa et al. 2008; Kumar et al. 2013a, b, c; Kumar et al. 2014a, b; Kumar et al. 2015a, b; Prasad et al. 2013). Appropriate understanding of molecular mechanism regarding the TLR signaling engenders us to investigate methods that modify this inflammatory response to evade corneal cell destruction caused by immoderate inflammation. Previous studies showed that among all TLR, TLR2 may have a great role in innate and acquired immunity. During normal TLR signaling, cytoplasmic TIR domain of TLR associated with adaptor protein MyD88 stimulates activation of downstream kinase enzymes and transcription factors such as NF-κB (Bowie and O’Neill 2000). It has been examined that profilin-like protein from *Toxoplasma gondii* acts as a first ligand for TLR11 that has been described as an important clue regarding the gene expression studies on mice but not on human. MyD88 plays an essential role in TLR signaling and is involved with TLR expression in eye (Lauw et al. 2005). TLR3 induces cytokine production through a signaling pathway dependent on MyD88 after activation with poly (I:C) and can induce production of NF-κB and mitogen-activated protein (MAP) kinases independently of MyD88 (Alexopoulou et al. 2001). Corneal inflammation (Keratitis) of eye is common problem, has been divided into major categories like amoebic keratitis, bacterial keratitis, fungal keratitis, and viral keratitis on the basis of pathogens associated with these (Fig. 1).

Amoebic keratitis (AK) is a painful and most serious corneal infection, caused by strain of protozoan—*Acanthamoeba*, detected in US in 2001. *Acanthamoeba* was first identified as an ocular pathogen and has been reported as a major cause of AK keratitis in 1980 due to contaminated contact lenses (O’Day and Head 2000). *Acanthamoeba* has been found in fresh water, sea water, air, soil and air condition system (El-Sibae 1993). Diagnosis of *Acanthamoeba* infection is a difficult task (Claerhout et al. 2004). This may be because several biocides are ineffective in killing metabolic inactive cyst stage of *Acanthamoeba*. Percentage of patients infected with AK due to wearing contact lens and soft contact lens was 80 and 75 %, respectively (Moore et al. 1985). It has been observed that immunization plays an essential role in declining the prevalence of *Acanthamoeba* keratitis (Kumar and Lloyd 2002).

Bacterial keratitis is a serious vision-threatening problem associated with trauma or ocular surface disease (Musch et al. 1983; Benson and Lanier 1998). It is also a contact lens-associated problem (Dart 1988). Both Gram-positive and Gram-negative bacteria are responsible for bacterial keratitis. The most common bacterial pathogens are *Pseudomanas aeruginosa*, *Staphyloccus aureus* and *Streptococcus pneumonia.* Gram-negative bacteria, *Pseudomanas aeruginosa*, are a major cause of bacterial keratitis in Unites States and have been found as a risk factor in contact lens wearer (Bourcier et al. 2005; Green et al. 2008). *Pseudomanas aeruginosa* is found to be ubiquitous in environment (Green et al. 1974). Gram-positive bacteria, *Staphyloccus aureus*, accounted for one quarter of confirmed cases in China and US (Sun et al. 2004a; Alexandrakis et al. 2000). *Staphyloccus aureus* is usually found in skin flora and anterior nares of nasal passages (Kluytmans et al. 1997; Cole et al. 2001). Some studies reported that 83 % cases of bacterial keratitis have been caused by Gram-positive bacteria like *Staphylococcus* species, which have been found in contact lens wearers. Gram-negative bacteria, mainly *Pseudomanas aeruginosa,* are responsible for 17 % cases of all bacterial keratitis in contact lens wearers. Overall, percentage contribution of Gram-positive, Gram-negative bacteria and polymicrobial is 83, 17 and 2 %, respectively (Bourcier et al. 2005).

Fungal keratitis is the second most common cause of keratitis in China after bacterial keratitis (Xie et al. 2008). There are about 15 million fungal species present in environment (Hube 2009). Fungal pathogens comprise *Fusarium oxysporum, Aspergillus fumigates and Candida albicans*. Filamentous fungi like *Fusarium* and *Aspergillus* are crucial causes of keratitis in India and southern China (Bharathi et al. 2007; Xie et al. 2006). Fungal keratitis problem is correlated with vision loss. This problem can be due to use of broad spectrum antibiotics, steroids and use of contact lenses (Iyer et al. 2006). Center for disease control (CDC) reported that out of 318 cases of *Fusarium* keratitis, 94 % cases are involved with soft contact lens wear (Chang et al. 2006). *Fusarium* attaches firmly to lens and can easily penetrate soft contact lenses. So, *Fusarium* has ability to form a biofilm on lenses and is a major risk factor for keratitis development, and nature of the biofilm depends on the type of contact lens (Ahearn et al. 2008). *Fusarium* growing as a biofilm is more resistant to *antimycotics.*

In viral keratitis, viruses cause corneal alteration that ranges from benign, self-limited conjunctivitis to sight-threatening trauma. Viral keratitis can be divided into several categories such as herpes simplex keratitis (dendritic keratitis) and adenovirus keratitis. HSV is an enveloped double-stranded DNA virus and considerably causes ocular infection. Epidemic studies of ocular HSV infection showed that HSV-1 is a prominent cause of viral keratitis (Liesegang 2001). Stromal keratitis reactions generated in eye due to HSV infection are major causes of blindness in developed countries like USA (Streilein et al. 1997). Ocular infection of mice with herpes simplex virus (HSV) causes induction of certain chemical changes related with the production of cytokine and chemokine (Biswas and Rouse 2005).

### Status of keratitis in world

The incidence rate of microbial keratitis is relatively low in developed countries while it is high in developing countries. The World Health Organization (WHO) demonstrated that corneal blindness caused by microbial keratitis is emerging as a main cause of visual impairment after cataract and glaucoma (Resnikoff et al. 2004). In a study authorized by WHO, Southeast Asia Regional Office in New Delhi (WHO/SEARO) estimated that 6 million corneal ulcers occur annually in the 10 countries of Southeast Asia Region enclosing a total population of 1.6 billion. In India, it is estimated that there are approximately 6.8 million people who have vision less than 6/60 in at least one eye due to corneal diseases. It has been expected that the number of individuals with corneal blindness in India will increase up to 10.6 million by 2020 (Dandona and Dandona 2003). In disparity, in the United States, corneal infection was estimated as 2–11 per 100,000 over a 38-year study period in Olmsted County, Minnesota (Erie et al. 1993).

### TLR-linked amoeboic keratitis

Ren and Wu (2011) investigated mechanisms related with activation of TLR 4 signaling pathway after challenging Wistar mice with *Acanthamoeba*. They determined TLR 4–NF-Kβ and TLR4–ERK1/2 signaling pathway responsible for cytokine (NF-κB, p-IKβ and p-Erk1/2) production in cornea of Wistar rat using inhibitors like PDTC and U0126. Similar findings have been obtained by Ren et al. (2010) in human corneal epithelial cells challenged with *Acanthamoeba.*

### TLR-linked bacterial keratitis

It has been seen in various researches that modulation in TLR signaling occurs in corneal epithelium cells in several ways after infection with bacterial pathogens, responsible for bacterial keratitis. Studies shows a role of different types of TLR and mechanism associated with TLR-linked bacterial keratitis. Khatri et al. (2002) determined specific mediators like PECAM-1, MIP2 and TLR4 that are responsible for *Pseudomonas aeruginosa* endotoxin-induced keratitis in BALB/c, C3H/HeN mice. They measure stromal thickness, haziness and neutrophil recruitment by in vivo scanning confocal microscopy and immune histochemistry, respectively. Saint Andre et al. (2002) show that endosymbiotic *Wolbachia* play an important role in corneal inflammation induced by bacterial lipopeptides that activate TLR2/TLR6/MyD88 signaling in the cornea. Zhang et al. (2004a) examined TLR5–NF-κB mechanism responsible for induction of inflammatory response in HCE cells against *Pseudomonas aeuroginsa* infection. They determined IKβ-α phosphorylation and degradation, expression of IL-6, IL-8 in mRNA, and secretion using Western blotting, RT-PCR and ELISA, respectively. Johnson et al. (2005) demonstrated that activation of TLR 2, 4 and 9 receptors occurs through common adaptor (MyD 88) after treating with Pam3Cys, LPS or CpG DNA in mice. Kumar et al. (2006b) explored that TLR2 regulates hβD-2 expression in HCEC cell lines after challenging with *Staphyloccous* *aureus* or synthetic lipopeptide Pam3cys. Huang et al. (2007) showed the importance of TLR4 against *Pseudomonas aeruginosa*. Carlson et al. (2007) demonstrated the mechanism of regulation of neutrophil infiltration by corneal proteoglycans like keratocan, lumican during innate response after infecting kera−/− and lum−/− mice with LPS. Hara et al. (2009) examined whether hypoxia or contact lens wear alters TLR4 signaling pathways in SV40 HCEC cells or not and observed reduction in expression level of mRNA and protein in SV40 HCEC cultured cells by RT-PCR and ELISA, respectively. Chinnery et al. (2009) demonstrated that cells belong to myeloid lineage like macrophage and neutrophil, respond to microbial products like LPS/TLR4-induced corneal inflammation in bone marrow chimeras and c-fms conditional ablation mice (Mafia). Ghosh et al. (2009) show that TLR recognize PAMP molecules of microbe, which plays an important role in *Pseudomonas* keratitis. Huang et al. (2006) show that mice deficient in MyD88 (TLR signaling molecule) reduce immune response against *Pseudomonas aeruginosa* but have higher potential for systemic infection. Ito and Hamerman (2012) observed that both live and bacterial products can activate TLR in cornea that leads to chemokine production and neutrophil recruitment in corneal stroma. They also show TREM-2 function as a negative regulator in TLR signaling. Sun et al. (2013) displayed that some in vivo and in vitro studies show that TREM-2 (novel cell surface receptor) expressed on DC, microglia, osteoclast and macrophages cell lines, inhibit cytokine production through PI3K/Akt signaling pathway after *Pseudomaonas aeruginosa* infection in mice. Huang et al. (2006) showed that on the ocular surface, TLR4 with a cluster of differentiation 14 (CD14) and LPS-binding protein (LBP) has been reported to induce immune responses against Gram-negative bacteria mainly *Pseudomonas aeruginosa*. Hayashi et al. (2001) revealed that TLR5 is responsible for recognizing bacterial flagellin and results in activation of NF-κB and TNFα production. Hemmi et al. (2000) demonstrated that TLR9 mediates cellular response against CpG DNA of bacteria. Zhang et al. (2009a) demonstrated that LPS unresponsiveness of HCE might be due to deficient expression of MD2, essential component for LPS–TLR4 signaling.

### TLR-linked viral keratitis

Kariko et al. (2004) reported that TLR 3 is activated by cellular mRNA of virus. Kumar et al. (2006a) used inhibitor against TLR3-associated HSV-2 infection. Hayashi et al. (2006) observed increased cytokine level through activation of TLR3 and TLR4 in corneal epithelial cells by HSV-1 infection. Ueta et al. (2005) displayed that mRNA expression of IL-6,8 and TLR3 increased in HCEC cells after stimulation with poly(C). Matsukara et al. (2006) found that synthetic double-stranded RNA molecules increase expression of cytokines like IL-1β, GM-CSF, IL-6, chemokines and ICAM-1 through activation of transcription factor like NF-κB or IRF-3 in epithelial cells by knocking down the genes related to inflammation with siRNA treatment. Chintakuntlawar et al. (2010) examined that viral component/capsid of adenovirus induces corneal inflammation similar to intact virus. Yamamoto et al. (2002) displayed that TLR3 triggers the production of IFN-β in response to double-stranded RNA, in an MyD88-independent manner, through the adaptor molecule. Yamamoto et al. (2003) observed that TRIF/TICAM-1 TRAM/TICAM-2 is another adaptor molecule involved in the MyD88-independent pathway whose function is restricted to the TLR4 pathway. Kumar et al. (2006c) displayed that TLR3 plays an important role in viral infection when HCEC cells were treated with virus. Diebold et al. (2004) show that not only endosomal recognition of influenza genomic RNA by means of TLR 7 and MyD88 molecules, but genomic RNA of non-viral origin also well recognized by TLR7 and MyD88 molecules, can induce TLR7-dependent cytokine production. Tabeta et al. (2004) demonstrated that TLR9 and TLR3 play a major role in innate immune defense against virus, cytomegalovirus in mice. Bitko et al. (2004) showed that HCECs are the targets of viruses such as HSV-1, adenovirus and respiratory syncytial virus (RSV), TLR3 in HCECs may function as a sensor for detection of viral infection and for initiation of the antiviral response in the cornea.

### TLR-linked fungal keratitis

Hu et al. (2009) observed that TLR4 level increased in corneal epithelial cells after infecting with *Fusarium* *solani* fungus in BALB/c mice. Gao and Wu (2006) and Zhao et al. (2009b) elucidated that TLR2 and TLR4 receptor on corneal epithelial cell was recognized by fungi like *Aspergillus fumigates* and *Fusarium solani*. Jin et al. (2008) observed that TLR4 and TLR9 level increased in cornea after infection with *Fusarium solani*. Yuan and Wilhelmus (2010) showed that TLR 2 and 13 are involved in *Candida albican* infection using BALB/c and C57BL/6 mice. Sun et al. (2010) determined the role of IL-1R1, MyD88 and TLR4 in innate immune response against contact lens-associated *Fusarium* keratitis. Hua et al. (2010) and Tarabishy et al. (2008) showed that the level of TLR4 is increased during *Fusarium solani* keratitis in BALB/c mice and TLR4 also involved in controlling fungal infection during *Fusarium oxysporum* keratitis in C57BL/6 mice. Tarabishy et al. (2008) displayed that MyD88^−/−^ and TLR4^−/−^ mice, but not TLR2^−/−^ mice, have an initially increased *F. oxysporum* burden because of reduced fungal clearance in the cornea. In *C. albicans* keratitis, TLR-knockout mice are helpful to determine the effect of TLR2 and 4 in experiment yet, the severity of fungal keratitis in murine mutant strains was similar to that in wild-type control mice, more fungi were recovered after 3 days of infection from TLR2^−/−^ than from TLR4^−/−^ mouse corneas. The results of this study were similar to another study done by Villamon et al. (2004). Zhang et al. (2004b) showed that in *Oropharyngeal candidiasis*, the level of TLR2 and 4 increased after fungal infection in mice. MyD88 is also essential for TLR2 and TLR4 signaling, yet the role of TLR2 and TLR4 is not clear in the development of corneal opacification; TLR4^−/−^ mice have an impaired ability to clear the infection so TLR4−/− is important in eradicating fungal infection. Gao and Wu (2006), Zhao and Wu (2008), Guo et al. (2008), Guo and Wu (2009), Zhao et al. (2009a) and Hu et al. (2009) showed that TLR2 and TLR4 on corneal epithelial cells, keratocytes and leukocytes recognize fungi such as *Aspergillus fumigates* and *Fusarium solani*. Bellocchio et al. (2004) and Meier et al. (2003) elucidated that TLR2 and TLR4 are important in the host response to other filamentous fungi, such as *A. fumigatus* and to the pathogenic yeasts *C. albicans* and *Cryptococcus neoformans* (Table 1).

**Table 1:** List of different types of TLR with causal organisms

Causal organism		Type of TLR	References
Bacteria	*Pseudomonas aeruginosa*	TLR 9 silencing involved in reducing neutrophil infiltration	Huang et al. (2005)
		Endotoxin of PA involved in TLR4-dependent expression of PECAM-1 and MIP-2	Khatri et al. (2002)
		Flagellin of PA involved in TLR5–NF-Kβ pathway for corneal inflammation	Zhang et al. (2004a)
		TLR4 involved in host resistance during *Pseudomonas aeruginosa* keratitis	Huang et al. (2006)
	*Staphylococcus aureus*	Corneal inflammation mediated by TLR2 and MyD88 in resident epithelial cells	Sun et al. (2006)
		TLR2-dependent recognition of bacteria peptidoglycan	Dziarski and Gupta (2005)
		TLR 2 role in bacterial endophthalmitis	Kumar et al. (2010)
	*Streptococcus pneumoniae*	TLR2 and 4-associated host–bacterial interaction	Bourcier et al. (2005)
	Endosymbiotic *Wolbachia*	TLR 4-linked inflammatory response	Saint Andre et al. 2002
Protozoan	*Acanthamoeba*	TLR4-stimulated inflammatory responses in the cornea	Alizadeh et al. (2014)
Fungi	*Aspergillus fumigatus*	TLR2-associated cytokine and chemokine production	Guo et al. (2012)
		TLR2 and TLR4 initiate immune responses	Guo and Wu (2009)
	*Candida albicans*	TLR2, but not TLR4, triggers cytokine production	Gil and Gozalbo (2006)
Virus	*Herpes simplex virus*-*1*	Toll-like receptor 3 agonist poly (I:C)-induced antiviral response in human corneal epithelial cells	Kumar et al. (2006a)
		Induce TLR3, 9 and IL-6 secretion, involved in corneal cell inflammation	Hayashi et al. (2006)
		Induce expression of cytokines, interferon and TLR7 in HCEC	Li et al. (2006)
	*Human adenovirus*	Empty viral capsid involved in TLR 4-linked keratitis	Chintakuntlawar et al. (2010)

### Therapy for keratitis

Sun and Pearlman (2009) investigated that eritoran tetrasodium (E5564) inhibits CXC chemokine production in cornea by stimulation with LPS (TLR4) but not by Pam3cys (TLR2). Kumar et al. (2006c) show that TLR2 Ab decreases Pam3cys-induced hβD2 production and IL-6,8, TNF-α secretion. NF-κB inhibitor completely blocks pam3cys-induced hβD 2 expressions and partially blocked by P38 MAP kinase and JNK inhibitors. Effect of above inhibitors has been seen in HUCL cells challenged with Gram-positive bacteria like *Staphylococcus*. Huang et al. (2007) elucidated a role of ST2 (member of TLR/IL-1R superfamily) to decrease corneal inflammation by negatively regulating type 1 cytokine but positively regulates type 2 cytokines (IL-10) against *Pseudomonas aeruginosa* keratitis in BALB/c mice. Zhang et al. (2004a) used anti-TLR5 antibody and anti-flagellin antiserum against *Pseudomonas aeruginosa* infection in human corneal epithelium cell. Guo et al. (2012) determined that TLR2 siRNA treatment attenuates *Apergillus fumigatus* keratitis by suppressing corneal inflammation. Wilhelmus (2008) showed that application of drugs like vidarabine, trifluridine, acyclovir or gancyclovir heals within 1 week. Interferon monotherapy can be used against herpes simplex virus. *Acanthamoeba* keratitis usually affect contact lens wearer and to prevent this problem improvement in disinfecting solution has been done because Verani et al. (2009) shows that it is not necessary solution was contaminated with *Acanthamoeba* sometimes anti-*Acanthamoebic* efficacy of solution was not sufficient. Hara et al. (2009) proposed the effect of immunosuppressive drugs like glucocorticoids for alteration of TLR3 pathway in human corneal epithelial cells against viral infection. Chintakuntlawar et al. (2010) demonstrated that adenovirus infection of the cornea induces chemokine expression and consecutive infiltration by leukocytes through RGD (arginine–glycine–aspartic acid) by contact between viral capsid penton base and host cell integrins.

## Limitations of previous researches

In above studies, animals like rabbit and mice have been mostly used for studying different types of keratitis. There has been found several disadvantages of using animal models because of physiological differences between human and animal eye like corneal size, corneal thickness, arrangement of corneal collagen (Hayes et al. 2007), properties of corneal epithelial cells and amount of actin (Jester et al. 2005). In most of above researches, animal models and human corneal epithelial cells are used and possess many disadvantages.

In many research broad spectrum antibiotics like vidarabine, trifluridine, acyclovir or gancyclovir linked with several side effects/risks because broad spectrum antibiotics can disturb normal flora in eye and drugs can be associated with drug resistance by pathogenic bacteria.

## Conclusions and future perspective

Improvement in corneal cell culture models would be useful in pathogenesis of ocular diseases because it can reduce risk associated with killing of animals. Some researchers use primary cell culture of corneal epithelial cells and cell lines with long lifespan as in vitro models for ocular toxicology studies and to explore human corneal epithelial cell biology but it is difficult to cultivate primary human corneal epithelium because of paucity of accessible tissue So, SV 40-immortalized HCEC lines with properties that have resemblance with normal human corneal epithelial cells can be used in pathogenesis of keratitis problem. It also has been observed that artificial corneal epithelium cell under serum-free conditions can act as better model than normal corneal epithelium cell for ocular surface studies. These cell cultures are useful for studying gene regulation and tissue development studies.

Understanding complex mechanisms associated with TLR-linked corneal inflammation will be helpful to device new therapeutic approach to modulate immune responses associated with TLR. Since some studies show that understanding of molecular pathways of TLR and RIG/Mda 5, which activate immune response against antiviral infection, will lead to novel approach for treating antiviral infection. Small molecules have been used for better understanding of molecular basis of infective keratitis. Toll like receptor namely TLR7 and TLR 8 can independently mediate recognition of small compounds like “imidazoquinoline R-848” suggest possible redundancy in these receptors. Understanding the function and biology of the corneal LPS receptor complex may lead to novel therapies for the management of ocular Gram-negative bacterial infections has been seen.

RNAi pathway is often providing good advantage to investigate function of genes in cell culture studies according to previous researches. RNAi is fast, uncomplicated and reliable effort to repress targeted genes expression. Therapeutic potential of RNAi has been revealed in several diseases like viral infection, hepatitis and ocular neovascularization. siRNA technique provides great advantage due to facile delivery of siRNA on cornea. It also has major applications in gene knockdown, functional genomics, medicinal field and biotechnology field studies. RNAi drugs show better response than antisense RNA molecules and antibody-based drugs. RNAi may be more effective than antisense RNA in human cancer cell lines. To deal with antibiotic resistance bacteria problem, some bacteria can be used to combat with drug-resistance bacteria. Predator bacteria like *Micavibrioaeruginosavorous* and *Bdellovibrio* *baceriovorous* may be susceptible to attack pathogenic MDR bacteria. These good bacteria can combat with bad bacteria.
